# Geospatial Patterns of Antimicrobial Resistance Genes in the US EPA National Rivers and Streams Assessment Survey

**DOI:** 10.1021/acs.est.2c00813

**Published:** 2022-06-23

**Authors:** Scott P. Keely, Nichole E. Brinkman, Emily A. Wheaton, Michael A. Jahne, Shawn D. Siefring, Manju Varma, Ryan A. Hill, Scott G. Leibowitz, Roy W. Martin, Jay L. Garland, Richard A. Haugland

**Affiliations:** ^†^Center for Environmental Measurement and Modeling and ^‡^Center for Environmental Solutions and Emergency Response, US Environmental Protection Agency, Cincinnati, Ohio 45268, United States; § Center for Public Health and Environmental Assessment, US Environmental Protection Agency, Corvallis, Oregon 97333, United States

**Keywords:** antimicrobial resistance, rivers, streams, anthropogenic pollution

## Abstract

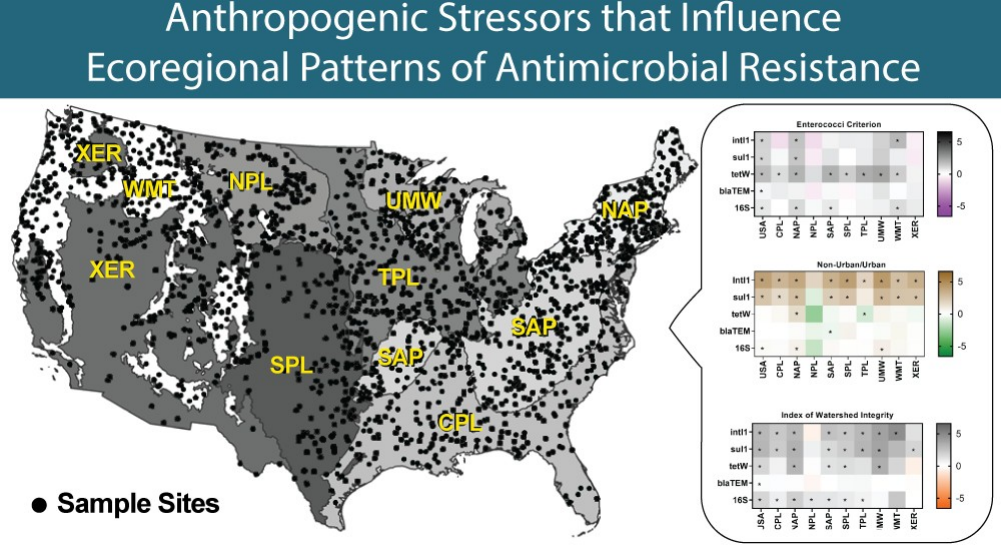

Antimicrobial resistance (AR) is a serious global problem due to the overuse of antimicrobials in human, animal, and agriculture sectors. There is intense research to control the dissemination of AR, but little is known regarding the environmental drivers influencing its spread. Although AR genes (ARGs) are detected in many different environments, the risk associated with the spread of these genes to microbial pathogens is unknown. Recreational microbial exposure risks are likely to be greater in water bodies receiving discharge from human and animal waste in comparison to less disturbed aquatic environments. Given this scenario, research practitioners are encouraged to consider an ecological context to assess the effect of environmental ARGs on public health. Here, we use a stratified, probabilistic survey of nearly 2000 sites to determine national patterns of the anthropogenic indicator class I integron Integrase gene (*intI1*) and several ARGs in 1.2 million kilometers of United States (US) rivers and streams. Gene concentrations were greater in eastern than in western regions and in rivers and streams in poor condition. These first of their kind findings on the national distribution of *intI1* and ARGs provide new information to aid risk assessment and implement mitigation strategies to protect public health.

## Abstract 2

Antimicrobial resistance gene concentrations are higher in US rivers and streams with disturbances in ecological function.

## Introduction

The discovery of antimicrobials from natural bacteria and fungi some 80 years ago ushered in a remarkable era of reduced morbidity and mortality due to human infections. Unfortunately, this medical innovation has led to the overuse of antimicrobials and contributed to the evolution of clinical antimicrobial resistance genes (ARGs) and increased spread of antimicrobial resistance (AR) bacterial (ARB) infections.^1^ Consumption rates of medically relevant antimicrobials for human health and animal food production in the US demonstrate geographic heterogeneity, with hot spots in the Midwest and Southeast regions and lower rates in the Northeast and Western regions.^2,3^ Due to the spread of ARG, 2.8 million individuals develop AR infections annually, resulting in 35000 deaths in the US.^4^ These infections result in $21–34 billion in health care annual costs and approximately 8 million additional days of hospital care.^6^ The annual cost to treat infections by six multidrug-resistant pathogens alone ranges from $4.1 to $5.1 billion.^7^

Urbanization and agriculture have led to increased AR in watersheds, which is concerning from a One Health (i.e., human, animal, environment) perspective because ARGs are capable of dissemination and proliferation within ARB by horizontal gene transfer, mutation and recombination, and selection.^8,9^ Rivers and streams near wastewater treatment plants, animal feeding operations, or sources of manure and municipal biosolids exhibit elevated ARGs associated with the use of antibiotics, implying that selection and/or wastewater discharge is contributing to the dissemination of these contaminants in aquatic resources.^10,11^ Elevated quantities of *intI1*, which is involved in the mobility of ARGs and has been used as a proxy indicator of anthropogenic pollution, and ARGs in individual watersheds have been associated with impaired ecological conditions, urbanization, agriculture, and fecal pollution.^12−16^ Bacterial community biofilms grown in artificial streams exposed to high nutrient loads in combination with antimicrobials (e.g., sulfonamides) showed significant increases in *intI1* and *sul1*, suggesting that a synergistic selective pressure influences their persistence in streams.^17^ Considering these observations, we hypothesize that *intI1* and ARG abundances are greater in rivers and streams affected by multiple anthropogenic stressors and that *intI1* can be used as a condition indicator. To explore this hypothesis, we utilized samples collected in the EPA’s National Rivers and Streams Assessment (NRSA) 2013–2014 survey, one of four periodic EPA surveys used to assess the quality of the nation’s aquatic resources.^18^ The samples were randomly collected in a probabilistic survey design to represent 1.2 million kilometers of perennial rivers and streams across the lower 48 states. Each survey site has an associated weight that corresponds to the length of stream represented by the site.

## Materials and Methods

### Sample Site Selection

**Figure 1:**
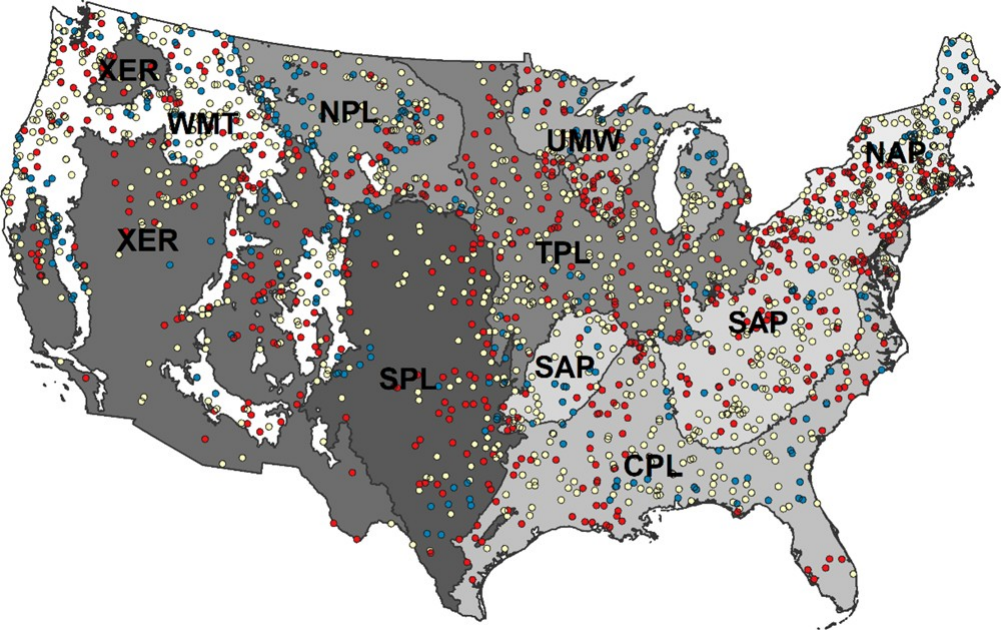
Sample sites for the 2013–2014 National Rivers and Streams Assessment survey. The nine aggregated Omernik Level 3 ecoregions are shaded. Abbreviations: Coastal Plains (CPL), Northern Appalachians (NAP), Northern Plains (NPL), Southern Appalachians (SAP), Southern Plains (SPL), Temperate Plains (TPL), Upper Midwest (UMW), Western Mountains (WMT), and Xeric (XER). There were 306 LDS (blue), 1105 IDS (yellow), and 641 MDS (red).

The US EPA’s NRSA is a survey designed to assess biological and recreational conditions using indicators of the condition and stress. The spatially balanced, probability-based design permits assessment at several spatial scales, including national, ecoregional, and state scales. Ecoregions are zones that share geographical and environmental characteristics (i.e., climate, geology, soil type, etc.).^18^ Samples collected under this design are representative of streams during low-flow conditions within the conterminous US and can be used to make statistically valid inferences on stream conditions within regions.^19^ The sample sites are spatially distributed across the lower 48 states and nine aggregated Omernik ecoregions (AG_ECO9) and include small streams (0–2 Strahler order), large streams (3–4), and major rivers (5 and above). The nine aggregated ecoregions are Coastal Plains (CPL), Northern Appalachians (NAP), Northern Plains (NPL), Southern Appalachians (SAP), Southern Plains (SPL), Temperate Plains (TPL), Upper Midwest (UMW), Western Mountains (WMT) and Xeric (XER) (Figure 1). Details of the sample selection process are described elsewhere.^18^ The 2013–2014 survey includes 1853 sites (Figure 1); each site has an associated weight that corresponds to the length of stream represented by the site. In total, the survey sites represent approximately 765000 miles, or 1.2 million kilometers, of US rivers and streams. In addition to the survey sites, 199 hand-selected sites in least disturbed conditions were sampled. These sites were used along with randomly sampled survey sites to establish regionally relevant benchmarks to assess ecological conditions.

### Categorization of Sample Sites

Sites were categorized by five condition classes: level of disturbance, catchment integrity, watershed integrity, fecal pollution, and urbanization. The details of these condition classes are described below. Least disturbed sites (LDS), intermediately disturbed sites (IDS) or most disturbed sites (MDS) were determined using six chemical (total phosphorus (TP), total nitrogen (TN), chloride, sulfate, acid neutralizing capacity, and dissolved organic carbon) and three three physical (turbidity, riparian disturbance index, and percentage of fine substrate) parameters. The parameter thresholds that define LDS and MDS are ecoregion-specific (Table S1). For a site to be considered a LDS, all LDS criteria need to be met. However, only one MDS parameter needs to be met for a site to be considered a MDS. All other sites are considered IDS. This condition class is referred to as “LIM”.

The sites were also classified using the index of catchment integrity (ICI) and index of watershed integrity (IWI). Flotemersch et al.^20^ proposed a reference-free framework that defines watershed integrity as the capacity of a watershed system to support and maintain a full range of ecological processes and functions essential for biodiversity and ecosystem services. The indices of watershed and catchment integrity numerically signify this framework, ranging from lesser (0) to greater (1) integrity. The ICI describes the integrity of six ecological functions in response to anthropogenic landscape stressors for the area contributing directly to a stream segment, whereas the IWI considers upstream conditions, including local and upstream sources of surface water.^21,22^ Geometric means of the ICI and IWI were used to classify the sites as good (above the geometric mean: ICG, IWG) or poor (below: ICP, IWP). Enterococci gene copy estimates were converted to calibrator cell equivalents (CCE) as described in EPA Method 1609.1 for comparison with the human-health-related statistical threshold value of 1280 CCE per 100 mL^23^ and used to classify the sites as above this threshold criterion value (AC) or below (BC). In addition, the sites were classified as urban (URB) and nonurban (NURB) using a 2015 urban area cartographic boundaries shapefile (label NRS13_URBN; https://www.census.gov/geo/maps-data/data/cbf/cbf_ua.html).

### Sample Collection and Filtration

At each NRSA site, water was collected into 250 mL sterile bottles at approximately 1 m from the bank and 12 in. below the water’s surface. A sodium thiosulfate tablet was added to neutralize chlorine. Sampling occurred May–October 2013 and 2014 as has been previously described.^24,25^ Samples were transported on ice, and duplicate 50 mL volumes were filtered through 0.45 μm polycarbonate filters using negative pressure. Membrane filters were then rinsed twice with 10 mL of chilled phosphate buffered saline (PBS) and then aseptically transferred to sterile tubes containing siliconized white ceramic beads (Green Beads, Roche) and placed on dry ice. Filtration and storage on dry ice occurred within 6 h of sample collection; longer term storage was maintained at −70 °C. Field blank samples were performed at approximately 10% of the sites by filtering 10 mL of the chilled PBS and transferring membrane filters to bead tubes that were stored on dry ice with the NRSA site samples.

### Extraction of Genomic DNA

Genomic DNA was extracted from one of the replicate filtered samples using the procedure described in EPA Method 1609.1^26^ for analysis of enterococci and *E. coli* by qPCR followed by purification of a portion of the crude lysate for analysis of ARG, *intI1*, and 16S rRNA genes by droplet digital PCR (ddPCR). Briefly, each sample tube received 0.6 mL of AE buffer (Qiagen, Germantown, MD) supplemented with 0.2 μg/mL of salmon testes DNA (Sigma-Aldrich) as an extraction and qPCR amplification control and was subjected to bead milling in an eight-place bead beater (Biospec Products, Inc., Bartlesville, OK) at the maximum rate for 1 min. DNA was recovered in the supernatants by centrifugation at 12000*g* for 1 min followed by centrifugation of the transferred supernatants at 12000*g* for 5 min. A portion of the clarified supernatants (0.3 mL) was purified using the DNA-EZ RW 02 Kit (GeneRite, LLC., North Brunswick, NJ), according to the manufacturer’s instructions, eluting twice with 75 μL each time for a total volume of 150 μL. The unpurified portion of the clarified supernatant was used for qPCR analysis of *Enterococcus* spp. (enterococci) and *Escherichia coli* (*E. coli*) and to evaluate the process efficiency of salmon testes DNA, as described in EPA Method 1609.1. Samples were extracted in batches, which included positive filter extraction control samples (a predetermined concentration of *E. faecalis* and *E. coli* cells) and negative filter extraction controls (PBS).

### Genes Used in the Study

We included *intI1* since it is involved in the mobility of ARGs and has been used as a proxy indicator of anthropogenic pollution.^12,27,28^ Sulfonamide-resistant dihydropteroate synthase (Sul1) and tetracycline-resistant ribosomal protection protein (TetW) were included because *sul1* is linked to the ARGs of class 1 integrons and because sulfonamides and tetracyclines are classified as “Highly Important” by the World Health Organization.^29^ We selected *blaTEM* because penicillin drugs are “Critically Important”^29^ and are widely used to treat human infections.^30^*Klebsiella pneumoniae* carbapenem-resistant (KPC) β-lactamase, plasmid-borne phosphoethanolamine transferase (Mcr-1), and d-Ala-d-Ala ligase homologue (VanA) were included because they are emerging resistance determinants for the drugs of last resort.^29^ Genetic markers for fecal bacteria (23S rRNA) enterococci and *E. coli* and for total bacteria (16S rRNA) were included to determine the effect of fecal and nutrient pollution on *intI1* and ARGs.

### Measurement of Enterococci and *E. coli* by qPCR

The detection of enterococci and *E. coli* 23S rRNA gene sequences was performed in duplicate using previously described qPCR assays (Table S2). Briefly, the target sequences were amplified in 25 μL reactions containing 5 μL of the sample, 100 copies of the internal amplification control (IAC) template, 1 μM each of forward and reverse primers, 80 nM each of target and IAC probes (Table S2), 5 μg of bovine serum albumin (Sigma-Aldrich), TaqMan Environmental Master Mix (Thermo Fisher Scientific, Microbiology Division, Lenexa, KS), and PCR-grade water. Reactions were run in a StepOnePlus Real-Time PCR System (Applied Biosystems, Foster City, CA) at initial denaturation at 95 °C for 10 min followed by 40 cycles of 95 °C for 15 s and 60 °C (56 °C for *E. coli* analyses of 2014 samples) for 1 min. Fluorescence thresholds were set at 0.03 ΔRN, and baseline cycles were determined using the AUTO feature in the instrument software. The salmon testes DNA was amplified using the Sketa22 assay (Table S2) as described above to detect PCR interference and normalize for DNA recovery. Quantification of target gene sequences was performed using a composite standard curve model with Sketa22 assay adjustments for DNA recovery in a prototype of the Excel workbook described by Lane.^31^ Plasmid DNA standards with multiple target sequences and estimated copy numbers ranging from 30957 to 10 per reaction^32^ were used to generate standard curves with *r*^2^ values >0.99.

### Measurement of *intI1*, 16S rRNA, and ARG using ddPCR

Droplet digital PCR was performed with the QX200 Droplet Digital PCR System (Bio-Rad Laboratories, Inc., Hercules, CA). Each duplex reaction consisted of 5 μL of the sample, ddPCR Supermix for Probes (no dUTP), 900 nM of the respective primers and 250 nM of the respective probes (Table S2) in a total volume of 25 μL. The ddPCR assay targets were *intI1*,^33^*sul1*,^34^*tetW*,^35^*blaTEM*,^30^*mcr-1*,^36^*blaKPC*,^37^*vanA*,^38^ and 16S rRNA gene.^39^ Droplets were generated using a QX200 Droplet Generator or an Automated Droplet Generator as instructed by the manufacturer. Except for the 16S rRNA gene assay, the PCR conditions consisted of 95 °C for 5 min, followed by 50 cycles of 95 °C for 30 s and 55 °C for 1 min, and a final incubation at 98 °C for 10 min. An annealing temperature of 60 °C was implemented for the 16S rRNA gene assay. Amplification was assessed with the QX200 droplet reader. Droplet fluorescence amplitude data were analyzed in R Version 3.2.3^40^ using the ddpcRquant package, as described.^41^ Extreme-value thresholding was based on combined data from *n* = 75–147 no-template controls (NTCs) across *n* = 25–27 PCR plates of each target, as described.^42^ Concentrations (mean molecules/droplet) were estimated by following a Poisson distribution through the Bayesian model to characterize the uncertainty of low-level detections.^42^ Sample densities (molecules/mL) were calculated on the basis of droplet volume (0.85 nL), sampled water volume, and a factor of 1500 to account for the proportion of original sample analyzed. The LLOQ for each target was defined as the upper 95% credible interval of estimated concentration in negative filter extraction controls; positive samples were those for which credible intervals did not overlap with the LLOQ. Quality control samples were included with every ddPCR plate. NTCs consisted of 10 mM Tris-HCl (pH 8.5, Qiagen). Positive controls were constructed by inserting target sequences into a custom gene (Integrated DNA Technologies, Coralville, IA) and linearized with *EcoRV* (New England Biosciences) according to the manufacturer’s instructions. The linear custom gene was quantified by ddPCR and diluted to 5 × 10^4^ molecules/μL for use as positive ddPCR controls. A second custom gene containing only the target sequence of the internal amplification control was digested with *Apa*I (New England Biosciences) according to the manufacturer’s instructions, quantified by ddPCR, and diluted to 5 × 10^4^ molecules/μL. The internal amplification control was composed of the corresponding DNA sequence of the Hepatitis G assay as previously described.^42^ Each sample was evaluated for potential PCR interference by adding 2 μL of IAC to ddPCR reactions and comparing the sample results to sample-free reactions.

### Assessment of Quality Controls

We implemented several types of quality control samples to monitor for false positive results. NTCs were included on every 96-well plate to determine whether contamination occurred during PCR analysis. PCR inhibition was evaluated using known concentrations of exogenously added DNA controls. Negative extraction controls were included with each extraction batch of samples to monitor for potential laboratory contamination during this process. Finally, field blanks were randomly collected at approximately 10% of the sites to assess the contamination introduced during sample collection and filtration in the field.

For the ddPCR analysis of ARG, *intI1*, and 16S rRNA, the NTCs (Table S3) were used to set a threshold for the classification of positive and negative droplets; all samples were used in this analysis. An assessment of the internal amplification control showed two samples with less than 50% recovery efficiency of the spiked control. These two samples were deemed inhibited and were excluded from further analysis. The negative extraction controls resulted in a 0–10.2% positivity rate for the various assays (Table S4) and were used to determine the LLOQ. The LLOQs were subsequently employed to classify the lengths and percentages of river and stream kilometers for all samples (Table S16) as described below in Mapping Regional Summaries. Using the LLOQ, the occurrences of *intI1*, *sul1*, *tetW*, *blaTEM*, *blaKPC*, *mcr-1*, and *vanA* were 54%, 49%, 34%, 7%, 0.9%, 0.1%, and 0.0%, respectively.

An analysis of field blanks showed a 0–7.4% positivity rate: 6 of 169 (3.5%) samples were above LLOQ for *intl1*; *sul1*, 12/169 (7.1%); *blaTEM*, 4/169 (2.4%); *mcr-1*, 0/169 (0%); *blaKPC*, 0/169 (0%); *tetW*, 7/167 (4.2%); *vanA*, 0/167 (0%). Although trace amounts of *intI1* and ARGs (log_10_ mean range 0.01–0.439) were present in the field blanks, these observations are unlikely to reflect the national condition of watersheds^22^ or outpatient^44^ and livestock^2^ antimicrobial consumption rates. To understand if field blanks introduce bias on the sample measurements, categorical predictive modeling was performed using the ecoregion as a sole predictor of *intI1*, ARG, and 16S rRNA distributions as described in Data Analysis. This analysis showed no associations between the ecoregion and any of these gene targets (Bayes factors for the ecoregion models were less than 1; Table S5). In contrast, for the river and stream samples there were decisive associations between the ecoregion and these genes. Bayes factors were greater than 10^31^ for *intI1*, *sul1*, *tetW*, and 16S rRNA; for *blaTEM*, the factor was 498 (Table S6). There were discernible national patterns for the river and stream sites, but not for the field blanks. On the basis of this analysis, all river and stream samples were included in the survey.

For qPCR analysis of enterococci and *E. coli*, acceptance criteria reported by Sivaganesan^45^ for standard curve slope values as well as for positive and negative control Cq measurements and intra-assay variation (all sample analyses were performed in duplicate) were adopted. All sample batches passed the QA acceptance criteria for qPCR and inhibition and extraction negative and positive controls. Amplification efficiencies ranged from 93% to 96%, and intercept Cq values ranged from 37.28 to 37.47 for the enterococcus assay and from 92% to 95% and from 38.08 to 40.06 for the *E. coli* assay, as determined from the different composite standard curves. LLOQ Cq values were determined as described by Lane^31^ and ranged from 34.45 to 34.84 for the *Enterococcus* assay and from 35.95 to 37.94 for the *E. coli* assay. Samples that passed the QA criteria^26^ and had mean Cq values >LLOQ were used for subsequent data analyses. Samples giving no detection of target sequences (0.86% and 0.48% of the samples for enterococci and *E. coli*, respectively) and failing QA criteria were excluded from further analysis. An analysis of field blank samples revealed a positivity rate of 7.4% (16/196) for enterococci and 5.05% (5/196) for *E. coli*. Categorical predictive modeling using the ecoregion as the sole predictor demonstrated low Bayes factors (enterococci, 1.44; *E. coli*, 0.302; Table S5) for the field blanks, whereas the river and stream samples had Bayes factors greater than 10^14^ (Table S6). On the basis of this analysis, all survey sites were included in the study.

### Data Analysis

Reference conditions were established for five anthropogenic condition classes: (1) urbanization (Urban); (2) stream impairment (LIM); (3) water quality criterion for protecting human health (WQC), and indices of (4) watershed (IWI) and (5) catchment integrity (ICI). The geometric mean of gene concentrations for references and the most affected condition of each class were determined on national and ecoregional scales and within categories of NURB/URB, LDS/MDS, BC/AC, IWG/IWP, and ICG/ICP, and plotted in GraphPad Prism 7 (version 7.04, GraphPad Software, Inc.). A Spearman correlation analysis was also performed in GraphPad Prism 7. Bayesian estimation supersedes the *t* test (BEST)^46^ was implemented with log_10_-transformed gene concentrations to discern credible differences of the means between NURB/URB, LDS/MDS, BC/AC, IWG/IWP, and ICG/ICP. The online tool implements the Bayesian model described by Kruschke^47^ to estimate a probability distribution for the difference in means between two groups. For each comparison, the model was run with the default number of burn-in samples (20000) and samples (20000).

A Bayesian multiple linear regression was performed using JASP version 0.14.^48^ The following Bayesian multiple linear regression was used to assess the influence of longitude and latitude (Table S7) and ARG (Table S8) on *intI1* written as
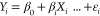
where *Y*_*i*_ is *i*th value of *intI1* or explanatory variables, β_0_ is the intercept, β_*i*_ are the coefficients for longitude and latitude or log_10_ ARG (*X*_*i*_; *sul1*, *blaTEM* and *tetW*), and ε_*i*_ is the residual error. The model prior was beta (1,1), the sampling method was Bayesian Adaptive Sampling (without replacement), and the number of samples for 95% credible intervals was 1000. The prior for the probability of inclusion (*P*_incl_) was Jeffreys–Zellner–Siow (JZS) that uses the Jeffreys prior on σ and the Zellner–Siow–Cauchy prior on the coefficients. We used the default squared *r* scale for JZS of 0.354. This regression compares two hypotheses: H_0_, the outcome variable is not predicted by the covariate(s), and H_1_, it is predicted by the covariate(s).

The nine ecoregions were used as categorical predictors for *intI1* and ARG while accounting for the 16S rRNA gene (Table S9). For this purpose, the BayesFactor R package implemented in JASP software was used to estimate the probabilities and parameters described below. The default value of 0.5 (i.e., *r* scale) was used for the shape parameter of the effect prior distribution. For numerical accuracy, we used 10000 draws from the posterior distribution to estimate BF. Models for the ecoregion levels were compared using the prior model odds (*P*_M_), the updated posterior probabilities after the model has seen the data (*P*_M|data_), the change in prior model odds after seeing the data (BF_M_), the BFs for each model (BF_10_), and the percent error due to Monte Carlo numerical fluctuations. The null model contained the 16S rRNA gene. The magnitude of the Bayes factor indicates the evidence for the rival hypotheses after having seen the data, which is the ratio of *P*_data|H1_ to *P*_data|H0_. The effects (ecoregions) were evaluated with the posterior probability of including the effect given the data (*P*_incl|data_), which is a sum of the posterior model probabilities (P_M|data_) that contain the categorical predictor and is interpreted as the evidence in the data supporting the inclusion of the condition class. Also, the Bayes factor of including the effect (BF_incl_) was monitored for the effects. Modeled averaged posteriors (Table S9) for the intercept and intercept-centered effects were compared by examining the magnitude of the 95% credible intervals. A *Q*–*Q* plot of the residuals was used to monitor whether the residuals approximated normality. Frequentist and Bayesian ANOVA were also performed in the absence of the 16S rRNA gene as a null covariate (Table S9). The same priors as described above were used and the model comparison BF_10_ values and the *P*_incl|data_ were reported for each gene. For ANOVA, partial η_p_^2^ and Type III Sum of Squares were used to estimate the effect size and the deviation from the mean value, and Kruskal–Wallis was used to determine if the samples were from the same distribution.

Five anthropogenic condition classes (e.g., Urban, LIM, WQC, IWI, and ICI) were used as categorical predictors for *intI1* and ARGs. We generated and ranked the models for each gene and monitored the BF_10_ for the models in comparison to the null. In addition, we monitored the posterior inclusion probability *P*_incl|data_ and the inclusion Bayes factor (BF_incl_) for the predictors. The inclusion probability is a sum of the posterior model probabilities (*P*_M|data_) that contain the predictor, and we interpreted it as evidence in the data in favor of including the predictors in the models. The following four predictor model scenarios were compared: (1) condition class, (2) condition class and ecoregion, (3) condition class, ecoregion, and 16S rRNA, and (4) condition class, ecoregion, and ARG normalized with 16S rRNA. Following the procedures above, the *P*_incl_, *P*_incl|data_, BF_incl_, and BF_10_ values for the best and next best models were included in the Bayesian analysis (Table S10).

Census regions (West, Midwest, South, and Northeast) were used as categorical predictors for years 2013 and 2014 US state outpatient prescriptions per 1000 persons (available at https://www.cdc.gov/antibiotic-use/data/outpatient-prescribing/index.html) and state-level weighted means of *intI1*, *sul1*, *tetW*, and *blaTEM*. JASP software was used for this analysis as described above. The default value of 0.5 (i.e., *r* scale) was used for the shape parameter of the census region prior distribution. We used automatic optimization for numerical accuracy and posterior samples to estimate BFs. Bayesian post hoc testing was performed for the census regions for each of these variables. For these comparisons, the posterior odds were corrected for multiple tests by fixing to 0.5 the prior probability that the null hypothesis holds across all post hoc comparisons.

### Mapping Regional Summaries

We generated state and ecoregion summaries with the spsurvey R package,^49^ including mean and median concentrations, total and percent of stream kilometers above the benchmark, and lower and upper 95% confidence intervals of each. We then used the ggplot2 R package^50^ to map and plot these summaries across the conterminous US. In addition, we mapped the percent of stream kilometers within ecoregions above the IWG-based benchmarks (i.e., above the upper 95% confidence interval of the geomeans) for *intI1*, *sul1*, *tetW*, and *blaTEM*. Pearson and Kendall correlations were performed between the kilometers for each gene using JASP version 0.14.1 (Table S11). A stretched β prior of 1.0 was used in the correlation analysis, and BF_10_ and 95% credible intervals reported the evidence for the alternative hypothesis relative to the null hypothesis.

### Guidelines for Data Generation and Reporting

The minimum information for publication of quantitative real-time PCR experiments (MIQE)^51^ guidelines checklist is given in Table S12. The minimum information for publication of quantitative digital PCR experiments (dMIQE2020)^52^ guidelines checklist is included as Table S13. The checklist for the environmental microbiology minimum information (EMMI) Guidelines^53^ is included in Table S14 The EMBRACE-WATERS (reporting antimicrobial resistance in waters) statement^54^ checklist is included in Table S15.

## Results and Discussion

### Geography of *intI1* and ARG in Rivers and Streams

**Figure 2:**
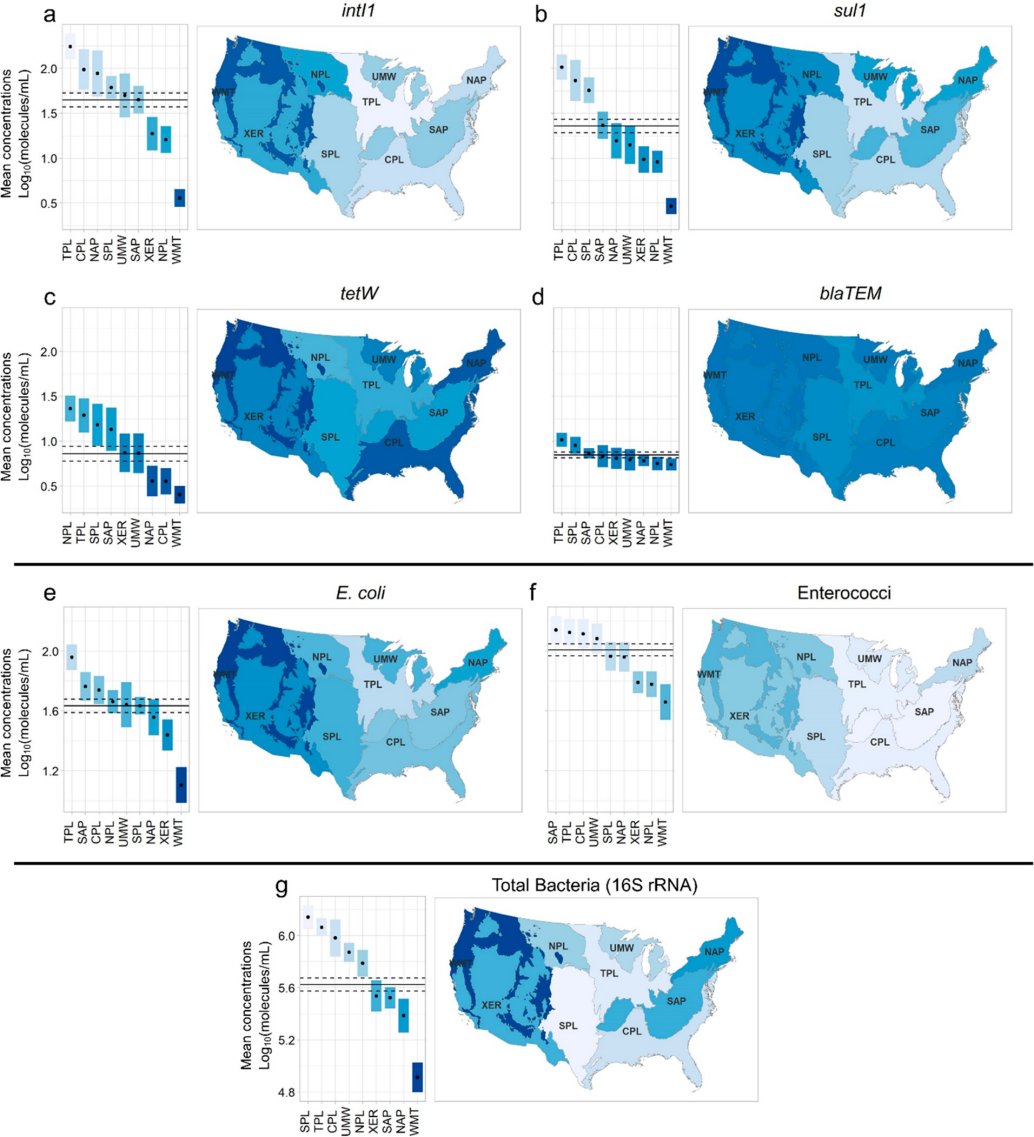
Geospatial distribution of genes among the nine ecoregions by weighted mean concentrations of (a) *intI1*, (b) *sul1*, (c) *tetW*, and (d) *blaTEM*, and ribosomal genes for (e) *E. coli*, (f) enterococci, and (g) 16S rRNA gene (total bacteria). The horizontal lines represent the national weighted mean and lower and upper 95% confidence intervals.

We investigated the geographical distribution of *intI1*, ARG, and fecal bacteria quantities in relation to nine ecoregions (Figure 1) with similar biotic and abiotic terrestrial and aquatic processes.^55^*IntI1* had the highest estimated mean concentration in rivers and streams at the national level, followed by *sul1*, *tetW*, and *blaTEM* (Figure 2 and Table S16). Enterococci amounts were higher than those of *E. coli*. Both *intI1* and *sul1* correlated with latitude and longitude (Table S7), and their concentrations were higher in the Plains, Appalachians, and Upper Midwest (UMW). *TetW* was higher in the Northern Plains (NPL), Temperate Plains (TPL), and Southern Plains (SPL). Estimates of state-level means of the genes, e.g., *intI1* and *sul1*, were consistent with the ecoregional pattern (Figure S1). National geospatial patterns were also demonstrated for enterococci and *E. coli* 23S rRNA genes and total bacteria 16S rRNA gene (Figure 2). In summary: (1) TPL and Coastal Plains (CPL) ranked near the top for most of the genes, (2) Xeric (XER), NPL, and Northern Appalachians (NAP) tended to be intermediate in rank rankings, and (3) Western Mountains (WMT) always ranked lowest.

### Environmental Drivers of *intI1* and ARG

We quantified the influence of fecal pollution on *intI1* and ARGs on national and ecoregional scales by performing a Spearman correlation analysis between the fecal bacteria 23S rRNA genes and *intI1* and ARGs. Weak correlations were observed between enterococci and *intI1* (USA, ρ = 0.13), *sul1* (ρ = 0.12), *tetW* (ρ = 0.25), and *blaTEM* (ρ = 0.06) (Figure S2 and Table S17). Low correlations were also observed between *E. coli* and *intI1* (USA, ρ = 0.30), *sul1* (ρ = 0.33), and *blaTEM* (ρ = 0.23). Moderate correlations were observed between *E. coli* and *tetW* (USA, ρ = 0.53) (Figure S2) among ecoregions that ranged from 0.23 for NPL to 0.60 for NAP. Previous studies have shown that tetracycline-resistant genes such as *tetW* are abundant in pig/human feces^56^ and culturable tetracycline-resistant *E. coli* are frequently detected at river sites near wastewater treatment plants (WWTP).^57^ The association between *E. coli* and *tetW* is consistent with the hypothesis that fecal pollution is an environmental driver of resistance to tetracyclines, which are widely used in food-production animals and human medicine.^29,58^

We next evaluated if these ARGs predict the presence of class I integrons. For this purpose, a multiple regression analysis was used to simultaneously quantify differences in model performances using all combinations of ARG as predictors of *intI1*. The model “*sul1*” was the best model (Table S8), while the models “*sul1* + *tetW*” and “*sul1* + *blaTEM*” were respectively 26.7- and 42.9-fold worse than the best model. There was a 909.1-fold reduction in performance when all three ARGs were modeled together, and models lacking *sul1* were indistinguishable from the null model. Poor model performances of *tetW* and *blaTEM* are not surprising, given that class 1 integrons are not known to contain *blaTEM*(59) or tetracycline-resistant gene cassettes.^60^ The best model “*sul1*” and the strong correlation between *intI1* and *sul1* (ρ = 0.78) is consistent with the location of *sul1* at the 3′-conserved segment of integrons,^61^ which is indicative of clinical class 1 integrons present in bacterial pathogens.^27,62,63^ Although this strong correlation suggests a linkage of these two genes, not all class I integrons contain *sul1*.^64^

We next considered the influence of the bacterial load on these genes because of the pronounced ecoregional variation of 16S rRNA genes (Figure 2). ARGs are often normalized by the 16S rRNA gene, and ARG/16S ratios are regarded as proxies for the proportion of bacteria-harboring resistance genes;^14^ these are used to distinguish the specific enrichment of ARGs from overall differences in bacterial levels.^15^ Enrichment of bacteria harboring ARGs may be due to imbalances in water chemistry: for instance, NO_3_^–^-N and Cl^–^ are known to affect river microbiome communities.^65^ Pruden et al.^14^ reported that 16S rRNA gene concentrations tended to be lower in the least disturbed rivers while they increased by factors up to 10 near animal feeding operations/WWTP. A strong correlation (ρ = 0.77) between 16S rRNA abundance and the percentages of river lengths in poor condition for total nitrogen (TN)^66^ underscores the importance of accounting for bacterial load. To account for the influence of bacterial load in the *intI1* and ARG geographical patterns, categorical predictive modeling was performed with ecoregions as fixed factors and 16S rRNA as a covariate in the null models (Table S9). There was strong statistical support for ecoregions explaining *intI1, sul1*, and *tetW* and evidence in favor of the *blaTEM* null model. The posterior means and credible intervals demonstrated agreement between *intI1* and *sul1* for all of the ecoregions except SPL and UMW. These analyses indicate an association between the ecoregions and *intI1*, *sul1*, and *tetW* that is not explained by bacterial load.

**Figure 3:**
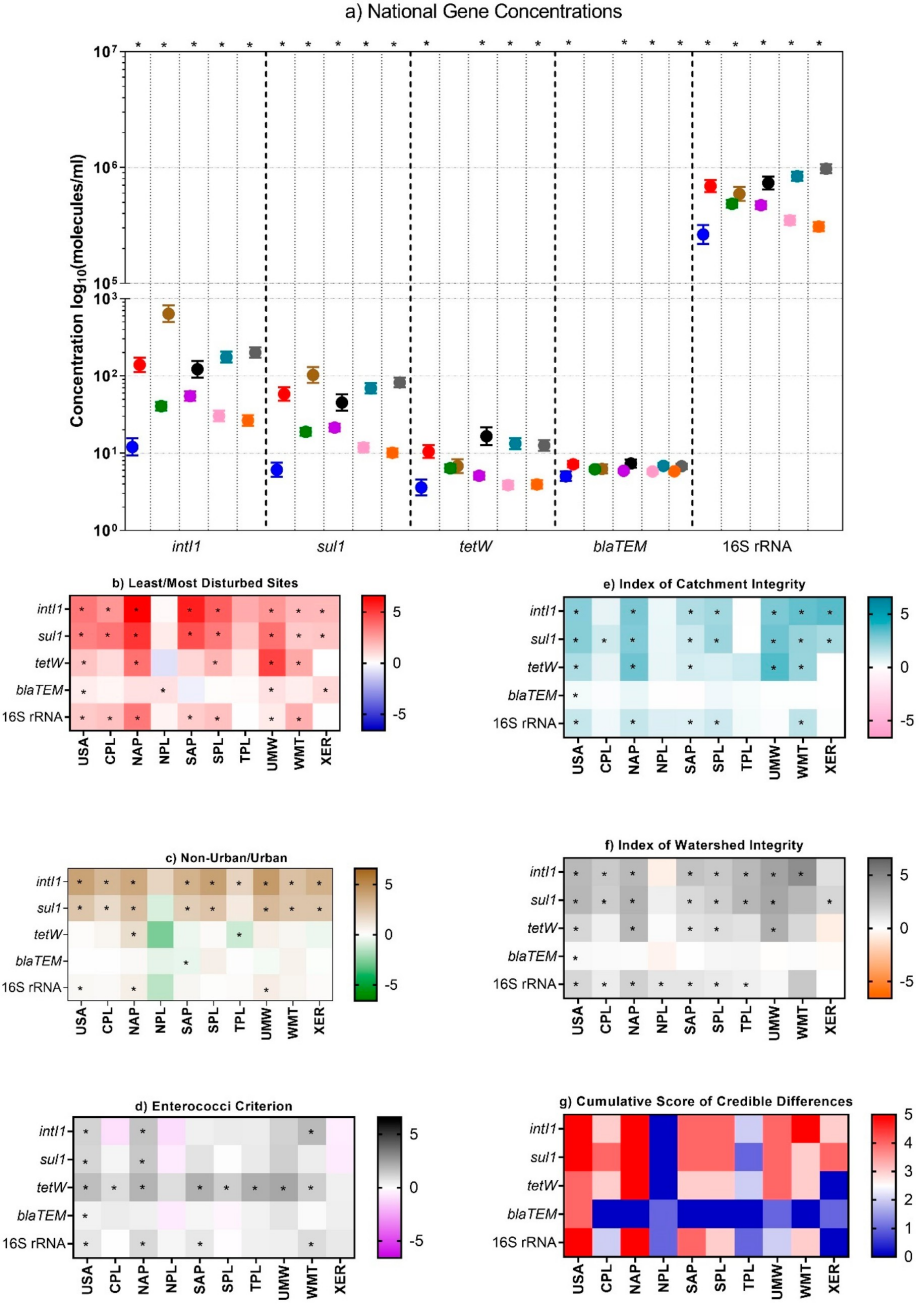
Comparison of gene concentrations using ecological condition (LDS/MDS), urbanization (URB/NURB), human health (AC/BC), ICI (ICG/ICP), and IWI (IWG/IWP) indicators. (a) National concentrations of *intI1*, *sul1*, *tetW*, *blaTEM*, total bacteria (16S rRNA) in LDS (blue circles), MDS (red circles), NURB (green circles), URB (brown circles), BC (orange circles), and AC (black circles). Heat maps of the log_2_ values of the ratios of (b) MDS to LDS, (c) URB to NURB, (d) AC to BC, (e) ICP to ICG, (f) IWP to IWG, and (g) cumulative score of credible differences among the condition classes. Asterisks in each box indicate 95% credibility of the difference of the means between the paired conditions.

Because AR is naturally occurring in the environment, it is important to consider background levels in each ecoregion in order to describe anthropogenic sources of AR.^67^ Comparisons between the baselines and poor conditions (i.e., LDS/MDS, URB/NURB, BC/AC, ICG/ICP, IWG, IWP) at the national scale indicated increases for *intI1* and *sul1* (Figure 3 and Table S18). Baseline estimates varied by ecoregion for each gene and five anthropogenic condition classes (i.e., Index of Watershed Integrity (IWI), Index of Catchment Integrity (ICI), recreational water quality criterion (WQC), Least, Intermediately, or Most (LIM) disturbed sites, and Urban (URB)). For example, using LDS as the baseline, reference conditions for *intI1* ranged from 3.5 to 50.2 molecules/mL (μ = 18.3, σ = 15.1, *c*_v_ = 82.5%). This is consistent with the expected variability between ecoregions due to different degrees of human activity and natural processes.^20^ There were increases in *intI1* and *sul1* in most of the ecoregions for all anthropogenic condition classes except for the WQC. Increases in national *tetW* and *blaTEM* occurred for all anthropogenic condition classes except for Urban. In addition, *tetW* increased for WQC in most of the ecoregions and in several ecoregions for LIM and Urban. Most ecoregions exhibited no credible *blaTEM* increases for any of the anthropogenic condition classes. In sum, 66.7%, 64.4%, and 46.7% of the *intI1*, *sul1* and *tetW* ecoregional comparisons, respectively, demonstrated increases in the anthropogenic condition classes whereas only 8.9% did for *blaTEM*.

We then treated the five anthropogenic condition classes as categorical predictors for *intI1* and ARG while controlling for the effects of ecoregion and 16S rRNA. Models were compared for each of the following scenarios: (1) classes alone, (2) classes controlling for ecoregion, (3) classes controlling for ecoregion and 16S rRNA, and (4) classes with 16S rRNA normalized *intI1* and ARG controlling for the ecoregion (Table S19). There was decisive support for scenarios 1–3, suggesting that IWI, LIM, and URB are associated with *intI1* and *sul1* and ICI, LIM, and WQC are associated with *tetW* (Figure S3). None of the classes in these scenarios were associated with *blaTEM*. When accounting for 16S rRNA as described in scenarios 3 and 4, we observed similarities for *sul1*, *tetW*, and, to a lesser degree, *intI1*. None of the classes explained *blaTEM* for scenario 3, whereas there was support for IWI and LIM in scenario 4. While ARG/16S ratios are regarded as proxies for the proportion of bacteria containing ARGs, an analysis based on gene ratio data may at times be nonintuitive, especially if environmental processes are differentially influencing the numerator and denominator.^68^ Although the inclusion of the covariates improved the predictive performance of the models, there remained a strong association between anthropogenic condition classes and gene abundances.

### Development of a Genetic Condition Indicator

The intricacies of identifying less-stressed watershed characteristics, as well as the ARG variability between ecoregion baselines (see Figure 3), underscore the challenge of establishing national standards for assessing the effect of anthropogenic contaminants across all watershed systems. A reference-free approach, such as ICI or IWI, is preferable because it does not rely on the challenging effort of identifying the characteristics and functions of theoretical pristine watersheds.^20^ Strong correlations of ICI and IWI with ARG (Figure S2) support using these indices for establishing ARG benchmarks. We selected IWI for this procedure because it summarizes the integrity for all local and upstream catchments.

**Figure 4:**
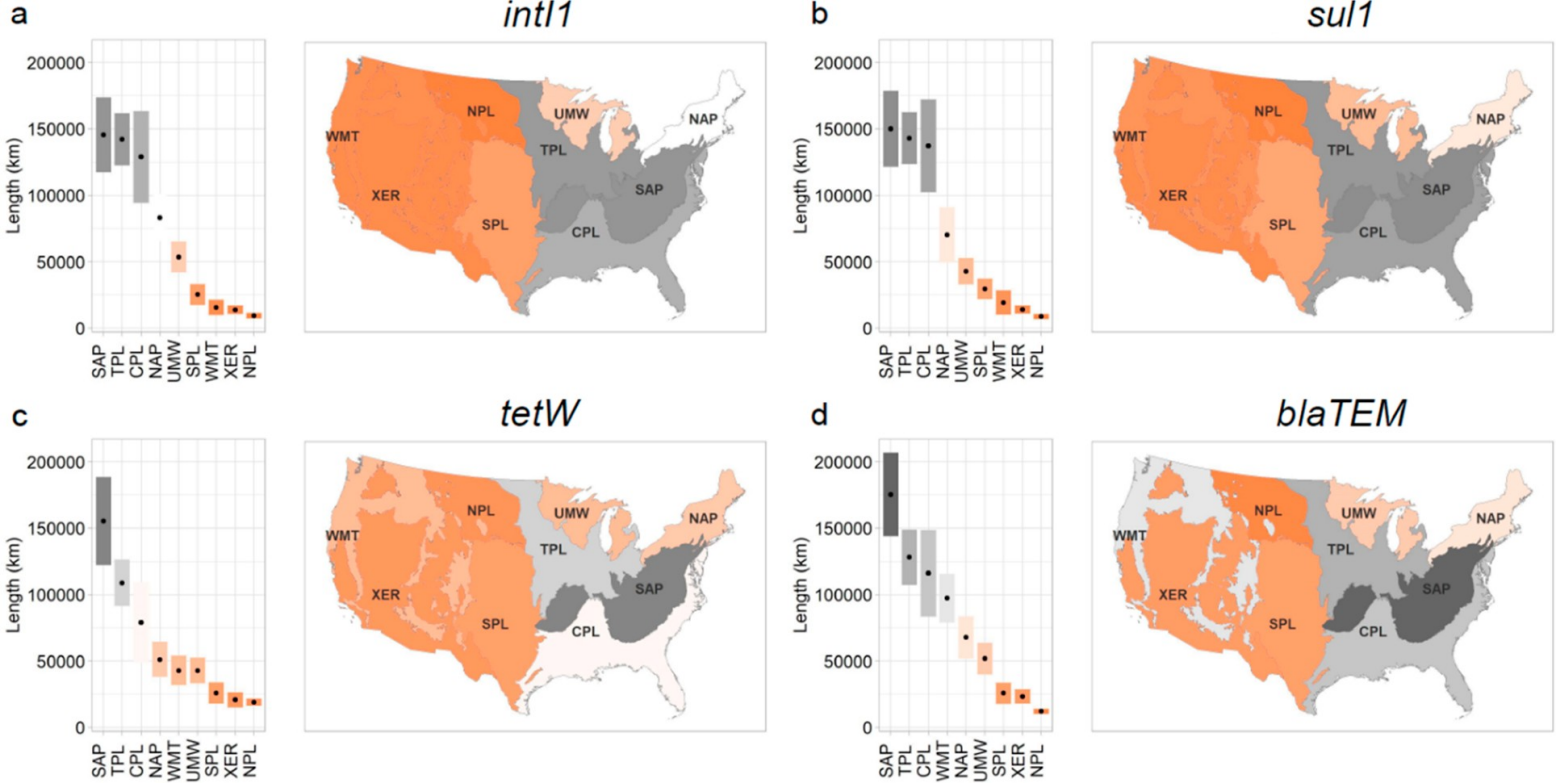
Geospatial distribution of targeted genes among the ecoregions by river and stream kilometers for sites where (a) *intI1*, (b) *sul1*, (c) *tetW*, and (d) *blaTEM* were detected above the reference IWG 95% threshold (Table S20).

The lengths of river and stream kilometers with *intI1*, *sul1*, *tetW*, and *blaTEM* concentrations above distribution-based IWI benchmarks (see Table S20) are shown in Figure 4, and the associated percentages of rivers and streams are shown in Figure S4. The ecoregion ranks for *intI1* and *sul1* were identical, with the kilometers exceeding the IWI benchmark ranging from 9279 (NPL) to 145622 (SAP) and 8765 (NPL) to 149983 (SAP), respectively. The numbers of kilometers for *intI1* and *sul1* were very similar to each other, as evidenced by a strong correlation (Kendall τ = 1.0, BF_10_ = infinity; Table S11), which is consistent with the genetic linkage of *intI1* and *sul1* in class 1 integrons.^61^ NPL and SAP also had the lowest and highest kilometers exceeding the IWI benchmark for *tetW* (18956, 155227) and *blaTEM* (12139, 175431), respectively. *TetW* and *blaTEM* kilometer ranks were similar to that of *intI1* for the top three ecoregions (SAP, TPL, CPL) and bottom ranks (XER, NPL). A correlation analysis indicated a strong association of *int1* with *tetW* (τ = 0.89, BF_10_ = 44.8) and with *blaTEM* (τ = 0.83, BF_10_ = 26.0). When they are taken together, the analyses provide evidence in favor of the *intI1* gene and ARG as indicators of anthropogenic condition for rivers and streams.

The more widespread occurrence of *intI1* and ARG in the SAP, TPL, and CPL ecoregions suggests that land use is a driver for AR. According to the EPA’s recent National Rivers and Streams Assessment (NRSA) Report,^18^ the habitat of SAP has been eroded by urbanization and agriculture, with 80% of its river/stream miles considered to be of poor quality due to total phosphorus (TP). Crop and animal agriculture are the primary land uses in TPL, with more than half (65.6% and 52.9%) of its river length classified as poor quality due to TN and TP. CPL has extensive bottomlands that are flooded for several months, and its habitats are affected by urban runoff. Finally, at least 37% of the river lengths of SAP, TPL, and CPL are above the human health criterion for enterococci. Interestingly, the similarity between the geography of *intI1* and ARG in these three ecoregions with outpatient and livestock antimicrobial consumption rates in the Midwestern and Southern regions of the US suggests that the use of antimicrobials contribute to the AR load.^2,3,44^ Outpatient antimicrobial prescriptions during years 2013 and 2014 were higher in the South census region (Table 1;^44^ 2013, 111.7 million; 2014, 110.5 million) and Midwest (61, 60.8) than in the West (47.0, 46.3) and Northeast (49.0, 48.6). To examine this hypothesis, we compared the distribution of US outpatient prescription rates with *intI1* and ARG concentrations. The prescription rates, *intI1*, and *sul1* were high in the Midwest, South, and Northeast, but they were lower in the West (Figure S5). *TetW* was higher in the Midwest and lower in the West. *BlaTEM* concentrations were similar across all regions. Post hoc comparisons for prescription rates, *intI1*, and *sul1* indicated decisive differences between the Midwest and South versus the West (Table S21).

The NRSA provides a statistical framework for examining spatial and, with data from future surveys, temporal variation of stressors and condition indicators in flowing freshwaters at national and regional scales. The results of our analyses are consistent with the hypothesis that anthropogenic stressors influence *intI1* and ARGs in US rivers and streams. Our baseline and model analyses indicate that the water quality (e.g., enterococci, total phosphorus, total nitrogen, etc.) and, more broadly, disturbances in ecological processes and functions essential for biodiversity and ecosystem services (captured by IWI/ICI) may play a role in promoting AR. Our results demonstrate that an ecological context is necessary to fulfill the One Health perspective of understanding the interconnectedness of AR in the environment. The geographic distribution of fecal bacteria (Figure 2) and AR (Figure 4) suggests that further studies should integrate these targets as microbial indicators of condition for public health analysis. Inclusion of a microbial component in the IWI could improve its sensitivity as an indicator of AR in One Health sectors (i.e., human, animal, environment) while better predicting rates of gastrointestinal illnesses.^69^ This report describes the first of an expected series of NRSA studies to monitor AR in rivers and streams in the US over time.

## Supporting Information Available

Heat map of *intI1* and *sul1* by state, Spearman correlation of ARG, FIB, ICI and IWI with ecoregions, Bayes factors from *intI1* and ARG models, distributions above the IWI impairment threshold, census region box plots of *intI1*, *sul1*, *tetW*, *blaTEM*, and outpatient prescriptions per 1000 persons, LDS and MDS parameters, PCR primers and probe assays, summary of negative template controls for ddPCR, LLOQ for ddPCR, Bayesian analysis of field blanks, Bayesian analysis of river and stream samples for comparison with field blanks, summary of linear regression analysis, *intI1* multiple linear regression results, model summary of gene abundance associations with ecoregions, effects and model analysis of condition classes, Bayesian correlation of genes and kilometers, MIQE qPCR, dMIQE2020, EMMI, EMBRACE-WATERS, concentrations above LLOQ and number and percent of stream kilometers above the LLOQ, gene correlations, baseline analysis results, inclusion probabilities and Bayes factor summaries, stream kilometers above benchmarks, and post hoc comparisons between census regions for *intI1*, *sul1*, *tetW*, *blaTEM*, and outpatient prescriptions per 1000 persons. (XLSX)

## Abbreviations

AC: above (recreational water quality) criterion
AG_ECO9: Nine aggregated Omernik ecoregions
AR: antimicrobial resistance
ARB: antimicrobial resistant bacteria
ARG: antimicrobial resistance gene
BC: below (recreational water quality) criterion
*blaTEM*: TEM-1 β-lactamase gene
CCE: calibrator cell equivalents
CPL: Coastal Plains
ddPCR: droplet digital PCR
FIB: fecal indicator bacteria
ICG: integrity catchment good
ICI: index of catchment integrity
ICP: integrity catchment poor
IDS: intermediate disturbed site
*intI1*: class I integron integrase gene
IWG: integrity watershed good
IWI: index of watershed integrity
IWP: integrity catchment poor
*blaKPC*: *Klebsiella pneumoniae* carbapenem-resistant β-lactamase gene
LDS: least disturbed site
LIM: combined least, intermediate, and most disturbed sites
*mcr-1*: plasmid-borne phosphoethanolamine transferase gene
MDS: most disturbed site
NAP: Northern Appalachians
NPL: Northern Plains
NURB: nonurban
SAP: Southern Appalachians
SPL: Southern Plains
*sul1*: sulfonamide-resistant dihydropteroate synthase gene
*tetW*: tetracycline-resistant ribosomal protection protein gene
TN: total nitrogen
TP: total phosphorus
TPL: temperate plains
UMW: Upper Midwest
URB: urban
*vanA*: d-Ala-d-Ala ligase homologue gene
WMT: Western Mountains
WQC: water quality criterion
XER: Xeric
16S: 16S rRNA gene
23S: 23S rRNA gene
